# OVA-Induced Food Allergy Leads to Neurobehavioral Changes in Mice and the Potential Role of Gut Microbiota and Metabolites Dysbiosis

**DOI:** 10.3390/ijms26104760

**Published:** 2025-05-16

**Authors:** Shouxun Hu, Chunyan Zhou, Yue Zhang, Luanluan Li, Xiaodan Yu

**Affiliations:** 1 Department of Developmental and Behavioral Pediatrics, Shanghai Children’s Medical Center, School of Medicine, Shanghai Jiao Tong University, Shanghai 200127, China; hushouxun@alumni.sjtu.edu.cn (S.H.); 13127715748@163.com (Y.Z.); liluanluan880829@163.com (L.L.); 2 Translational Medicine Institute, Shanghai Children’s Medical Center, School of Medicine, Shanghai Jiao Tong University, Shanghai 200127, China; zhouchunyan0318@163.com; 3 State Environmental Protection Key Laboratory of Environmental Health Impact Assessment of Emerging Contaminants, School of Environmental Science and Engineering, Shanghai Jiao Tong University, Shanghai 200240, China

**Keywords:** food allergy, neurobehavior, gut microbiota, metabolomics, amino acid metabolism

## Abstract

The neurobehavioral changes in food allergy mice have not been comprehensively studied, and the mechanism underlying them remains unclear. Our study aims to fully investigate neurobehavioral changes in OVA (ovalbumin)-sensitized food allergy mice and explore the potential mechanism via the gut microbiota–brain axis. We established the food allergy mouse (C57BL/6J male) model with OVA, evaluating the anaphylactic symptoms and the levels of Th2 signature cytokine and allergy-related antibodies in serum. Using behavioral tests, we measured anxiety, depression, social behavior, repetitive behavior, attention, and spatial memory in control and OVA mice. In addition, we analyzed the prefrontal cortex for measuring inflammation-related indicators and gathered serum for untargeted metabolomics analysis and feces for 16S rDNA sequencing. OVA mice exhibited anaphylactic symptoms and significantly elevated serum IgE and Th2 signature cytokine levels. In addition to anxiety-like, depression-like, and repetitive behaviors, OVA mice also displayed less social interest and damaged attention. TNF-α, IL-1β, and IL-6 levels and the activation of microglia in the prefrontal cortex of OVA mice were significantly increased, which might explain the neuronal damage. Using multi-omics technology, amino acid metabolism disruption, particularly carboxylic acids and derivatives, was observed in OVA mice, which was remarkably correlated with the altered abundance of gut microbiota related to food allergy. Behaviors in OVA-induced food allergy mice were extensively impaired. The disruption of amino acid metabolism associated with gut microbiota dysbiosis in OVA mice might play a pivotal role in impairing neural immune homeostasis and neuronal damage, which could be responsible for behavioral abnormalities.

## 1. Introduction

Food allergy (FA) is a complex heterogeneous inflammatory disease triggered by normally innocuous food protein intake and has gradually become one of the major problems threatening human health worldwide [1,2], particularly in developed countries. The current epidemiological data show that approximately 10% of people are afflicted with FA around the world [3,4], and the incidence of FA has increased remarkably in the past 2 to 3 decades [5,6]. At the same time, food allergies also impose a substantial economic burden on society and countries. In the USA, the 2013 report showed that the overall economic cost of food allergy was estimated at USD 24.8 billion annually (USD 4184 per year per child) [7]. Hence, preventing or finding more effective treatments for food allergies remains a formidable task for researchers, pediatricians, and physicians in future.

Generally, the typical symptoms of food allergy include disturbances to the skin, respiratory tract, and gastrointestinal tract, as well as cardiovascular aberrations, i.e., pruritus, urticaria, angioedema, abdominal pain, diarrhea, vomiting, and systemic anaphylaxis [8,9], whereas increasingly more clinical studies have reported the contribution of FA to emotional and behavioral dysfunction [10], like anxiety, depression, attention deficit hyperactivity disorder (ADHD) [11], and autism spectrum disorder (ASD) [12]. In alignment with these findings, our previous investigation also observed that infants with persistent allergy disease exposure at 6 and 12 months old, including food allergy, had a significantly increased risk for social-emotional development delay [13]. In addition, autism-, anxiety-, and depression-like behaviors have also been examined and well-verified with animal experiments or in food allergy rodent models. It should be noted, however, that ADHD-related behaviors in food allergy animal models have not been investigated. Collectively, food allergy might be relevant to all these abnormal behaviors, while these behaviors have not been systematically evaluated in food allergy animal models.

Gut microbiota (GM) is an assortment of microorganisms (bacteria, fungi, archaea, and viruses) inhabiting the length and width of the mammalian gastrointestinal tract, which is intimately involved in numerous aspects of human physiology, from nutritional status to behavior and stress response. Many studies have reported that the impaired homeostasis of GM causes food allergy [14] and neurodevelopmental disorders (anxiety, depression, ASD, and ADHD) [15], and some showed that probiotics intervention, such as *Lactobacillus* or *Bifidobacterium*, could effectively alleviate anxiety and depression [16,17,18,19] or reduce allergic inflammation and dampen Th2 response [20,21,22]. However, these previous studies only investigated the GM’s role in behavioral changes and in food allergies separately, and the role of the gut microbiota–brain axis in linking food allergy and neurodevelopmental disorders has not been studied, particularly with muti-omics technologies.

To fill this knowledge gap, we aimed to investigate the altered neurobehavior in a classical FA mouse model and explore the potential mechanism of the gut microbiota–brain axis between them via multi-omics techniques. A series of behavioral tests were performed in FA and control mice. Neuroinflammation was measured. In addition, fecal microbiomes and serum untargeted metabolomics were compared between two groups to detect the potential strains and metabolites involved in neurobehavioral changes.

## 2. Results

### 2.1. OVA-Induced Food Allergy Mouse Model

**Figure 1:**
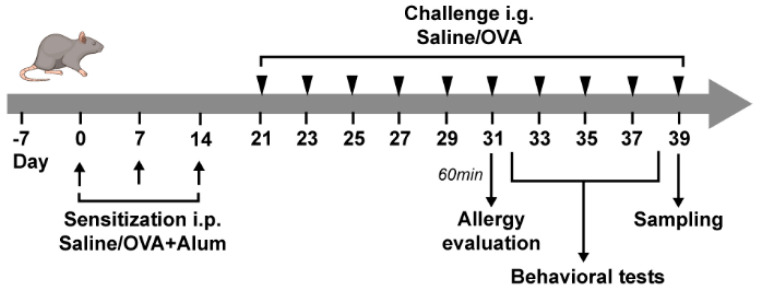
Schematic timeline of experimental procedure. OVA mice were sensitized by ovalbumin/aluminum and challenged by ovalbumin (OVA group). Control mice were treated with aluminum and saline only (CON group). At the end of treatment, mice were subjected to behavioral tests and then sacrificed for tissue and blood collection.

**Figure 2:**
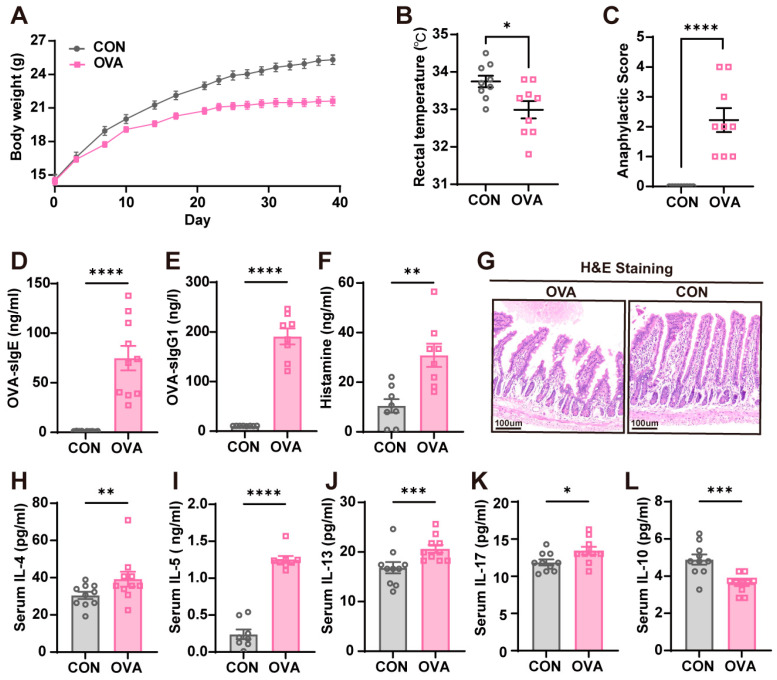
Confirming OVA-induced food allergy in mice. (**A**) Reduced body weight gains in OVA mice. (**B**) Reduced rectal temperatures in the OVA mice. (**C**) Increased anaphylactic scores in the OVA mice. (**D**–**F**) The OVA group showed an increased level of (**D**) OVA-specific IgE, (**E**) OVA-specific IgG1, and (**F**) histamine in serum. (**G**) Representative images of intestinal morphology by H&E staining. (**H**–**L**) The OVA group showed elevated levels of (**H**) IL-4, (**I**) IL-5, (**J**) IL-13, and (**K**) IL-17 and a deceased level of (**L**) IL-10 in serum. n = 8–10 in each group. Data are presented as the means ± SEM. ns = not significant. * *p* < 0.05, ** *p* < 0.01, *** *p* < 0.001, and **** *p* < 0.0001 using Student’s *t*-test or Mann–Whitney U test.

The experimental protocol is displayed in Figure 1. A mouse model orally induced with OVA (ovalbumin, one of the main allergens in eggs) allergy was successfully established (Figure 2). Compared to the control mice, the OVA mice exhibited slower body weight gains after repeated challenges (Figure 2A). After the last challenge, OVA mice also showed significantly higher anaphylactic scores and a lower core body temperature than the control mice (Figure 2B,C). As the main indicators for allergic response, OVA-sIgE and OVA-sIgG1 antibody levels in serum were measured, and OVA mice had significantly higher levels of OVA-IgE and OVA-IgG1 (Figure 2D,E). Histamine, the signaling chemical of allergy released by mast cells or basophil degranulation, also showed a significant elevation in the serum of OVA mice (Figure 2F). The morphological damages in the small intestines were evaluated with H&E staining, and intestinal sections in OVA mice showed mucosal atrophy and edema, epithelial detachment, and villous atrophy; no morphological damages were found in the control mice (Figure 2G). Lastly, aberrant Th2- and Th17-type responses with a significantly higher secretion of IL-4, IL-5, IL-13, and IL-17 were found in OVA mice (Figure 2H–K); contrarily, the levels of IL-10 which functioned as an anti-inflammatory response were suppressed in OVA mice (Figure 2L).

### 2.2. OVA Mice Exhibited Behavioral Abnormalities

**Figure 3:**
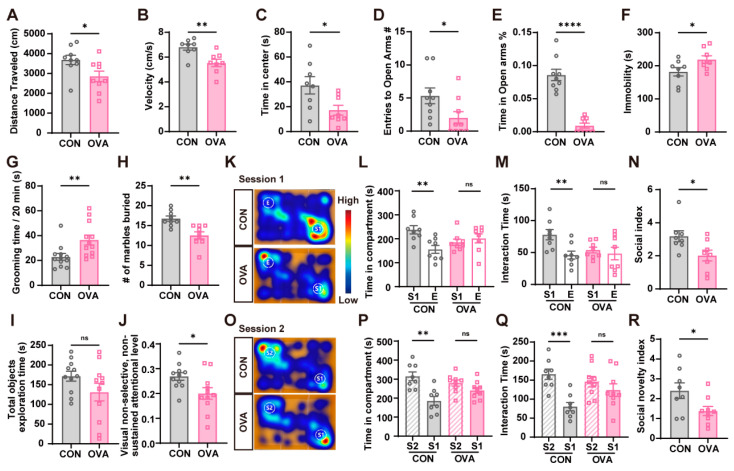
Extensive behavioral abnormalities induced by food allergy. (**A**–**C**) Open-field test (OFT): Reductions in (**A**) total traveled distance, (**B**) velocity, and (**C**) time spent in the central area in the OVA group compared to the CON group. (**D**,**E**) Elevated plus maze (EPM): OVA mice had a decrease in (**D**) entries into open arms and (**E**) the percentage of time spent in the open arms of the EPM. (**F**) Forced swimming test (FST): The immobility time was increased in the OVA group compared to the CON counterpart. (**G**) Self-grooming: The increased time spent on self-grooming in the OVA group. (**H**) Marble burying test: The buried marble numbers were reduced in the OVA group compared to the CON group. (**I**,**J**) Non-selective, non-sustained visual attention test (NNAT): (**I**) Mice in the two groups spent a similar time in total object exploration, and (**J**) the OVA group showed a decreased NNAT attention level. The NNAT attention level was calculated as [(time spent exploring new object)/(total time spent exploring objects)] × 100%. (**K**–**N**) Three-chamber sociability test (TST) in Session 1: (**K**) A representative trajectory heatmap of mice in Session 1. E: Empty cup, S1: Stranger mice 1. CON mice spent significantly more time in (**L**) the compartment of stranger 1 and (**M**) interactions with stranger 1 than of/with an empty cup, while no statistical significance was observed in OVA mice. (**N**) OVA mice showed a decreased social index, which was calculated as [(time spent interaction with stranger 1)/(time spent interaction with empty cup)] × 100%. (**O**–**R**) TST in Session 2: (**O**) A representative trajectory heatmap of mice in Session 2. S1: Stranger mice 1, S2: Stranger mice 2. CON mice spent more time in (**P**) the compartment of stranger 2 and (**Q**) the interaction with stranger 2 than of/with stranger 1, while no statistical significance was found in OVA mice. (**R**) OVA mice showed a decreased social novelty index, which was calculated as [(time spent interaction with stranger 2)/(time spent interaction with stranger 1)] × 100%. n = 8–12 in each group. Data are presented as the means ± SEM. ns = not significant. * *p* < 0.05, ** *p* < 0.01, *** *p* < 0.001, and **** *p* < 0.0001 using Student’s *t*-test.

After the challenge, a set of behavioral tests were conducted to evaluate the behavioral alterations in mice. Except for the spatial cognition in the Barnes maze test (Figure S1), the other behaviors were significantly altered in OVA mice (Figure 3). Firstly, OVA mice developed anxiety-like and depressive-like behaviors. In OFT, the control mice were more active than OVA mice as the former traveled longer distances and had higher velocity, while the latter spent less time in the central area (Figure 3A–C). Likewise, OVA mice spent less time in the open arms in the EPM test (Figure 3D,E), and had increased immobility time in the FST (Figure 3F). Next, compared to the control mice, OVA mice showed a significantly increased duration of repetitive self-grooming behavior (Figure 3G), and a reduced number of marbles buried, showing less digging activity related to food allergy (Figure 3H). OVA mice also showed impaired attention based on the NNAT (Figure 3J). In this test, although mice in two groups had the same total exploration time of objects after familiarization, OVA mice had a significantly decreased visual non-selective, non-sustained attention level compared to the control mice. Finally, the TST showed OVA mice with impaired social behavior and communication (Figure 3K–R). In the sociability test (Session 1), OVA mice were less interested in interacting with stranger mice compared to the control mice; consistently, in the social novelty preference test (Session 2), OVA mice showed greater apathy in interacting with stranger mice 2 than the control mice.

### 2.3. Food Allergy-Induced Neuroinflammation and Neuronal Damage

**Figure 4:**
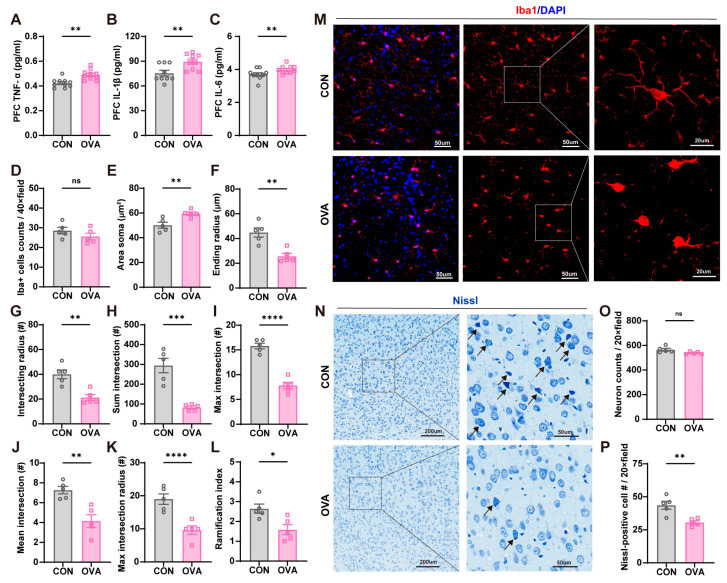
Neuroinflammation and neural damage in the PFC of OVA mice. (**A**–**C**) OVA mice showed elevated levels of (**A**) TNF-α, (**B**) IL-1β, and (**C**) IL-6 in the PFC, compared to the CON group. n = 9–10 in each group. (**D**) Similar microglia counts in the PFC between the OVA and CON groups. n = 5 in each group. (**E**) An increased soma area in OVA mice compared to CON mice. n = 5 in each group. (**F**–**L**) The microglial morphology towards an activated phenotype in the OVA group in terms of the following parameters extrapolated from Sholl analysis: (**F**) ending radius, (**G**) intersecting radius, and (**H**,**I**) sum and max intersection. The OVA-induced FA decreased the (**J**) mean intersection and (**K**) max intersection radius. (**L**) The reduced ramification index in the OVA group. n = 5 in each group. (**M**) Representative images of the morphological changes of microglia. (**N**) Representative images of the neuronal changes by Nissl staining. Arrows depict Nissl-positive cells. (**O**,**P**) Mice in the two groups had similar (**O**) neuron counts in the PFC, and the OVA group showed (**P**) fewer Nissl-positive cells. n = 5 in each group. Data are presented as the means ± SEM. ns = not significant. * *p* < 0.05, ** *p* < 0.01, *** *p* < 0.001, and **** *p* < 0.0001 using Student’s *t*-test.

As the prefrontal cortex (PFC) is an important region involved in memory, mood, and behavioral regulation [23], neuroinflammation and neuronal damage in the prefrontal cortex area of mice were measured (Figure 4). The pro-inflammatory cytokines, i.e., TNF-α, IL-1β, and IL-6, were remarkably increased in OVA mice compared to those in the control mice (Figure 4A–C). Microglia are immune cells surveilling the inflammatory state of the brain [24], and its number in the PFC did not differ significantly among the two groups (Figure 4D). Then, we further conducted Sholl analysis on the Iba1-positive cells to distinguish morphological changes and microglial activation in the two groups. We observed that in the PFC of the OVA mice, microglia cells showed a reduced morphological complexity of arborization, suggesting a significant polarization toward a reactive phenotype (Figure 4M). The detailed morphological changes in the OVA group included a significantly increased soma area and decreased ending radius, intersecting radius, sum intersection, max intersection, mean intersection, max intersection radius, and ramification index (Figure 4E–L). Neuronal injury assessment in the PFC showed no difference in total neuron counts between the two groups, but Nissl bodies significantly decreased in the OVA mice, indicating neuronal damage in the PFC induced by food allergy (Figure 4N–P).

### 2.4. Food Allergy Induced Gut Microbiota Dysbiosis

**Figure 5:**
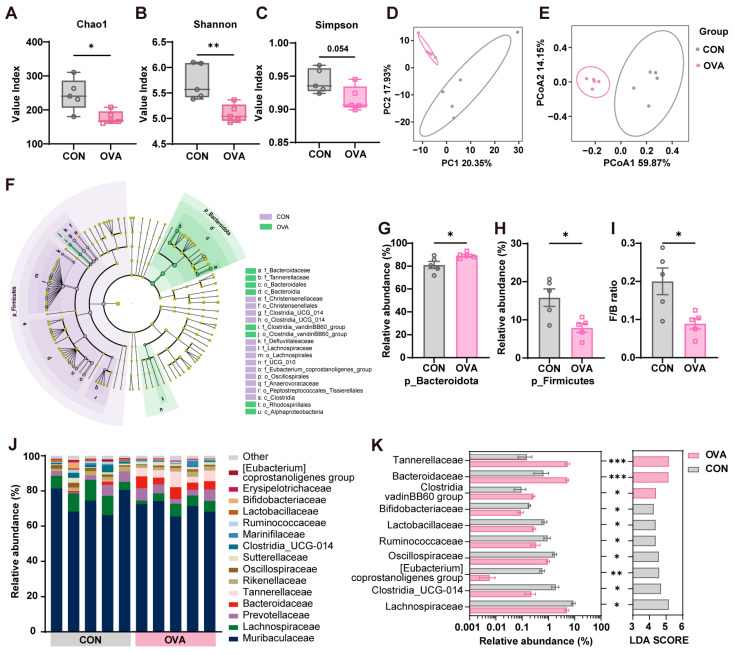
The disrupted balance of gut microbiota in OVA-induced FA mice. (**A**–**C**) The alpha diversity of the gut microbiota in OVA mice showed a decreased index including the (**A**) Chao1, species richness; (**B**) Shannon, species diversity; and (**C**) Simpson, species evenness. (**D**,**E**) Beta diversity analysis performed by (**D**) PCA and (**E**) PCoA. (**F**) LEfSe results in the OVA group and CON group. (**G**,**H**) The OVA group showed an increased relative abundance of (**G**) Bacteroidota and a decreased relative abundance of (**H**) Firmicutes at the phylum level. (**I**) A decreased ratio of Firmicutes to Bacteroidota in OVA mice. (**J**) The OVA group showed a different gut bacterial composition at the family level compared to the CON group. (**K**) The relative abundance and LDA score of 10 bacteria in 2 groups at the family level. N = 5 in each group. Data are presented as the means ± SEM. * *p* < 0.05, ** *p* < 0.01 and *** *p* < 0.001 using Student’s *t*-test.

16s rDNA sequencing was performed to evaluate the effects of FA on the gut microbiota in mice. In line with [25], the alpha diversity (Chao, Shannon, Simpson) was lower in OVA mice (Figure 5A–C), and the compositional dissimilarities in the beta diversity (PCA, PCoA) were significantly different in the two groups based on the multivariate cluster analysis (Figure 5D) or Bray–Curtis dissimilarity (Figure 5E). Figure 5F presents the LEfSe results in two groups and shows that the dominant bacteria at the phylum level were Bacteroidota and Firmicutes. In addition, the OVA group showed an increased abundance of Bacteroidota (Figure 5G) and decreased abundance of Firmicutes (Figure 5H). The ratio of Firmicutes to Bacteroidota also decreased in the OVA group (Figure 5I). Figure 5J shows the taxonomic abundance of the intestinal flora at the family level between the two groups, and 16 differential OTUs with relative abundance > 0.1% were filtered out. Compared to the control mice, the levels of *Bifidobacteriaceae*, *Lactobacillaceae*, *Ruminococcaceae*, *Oscillospiraceae*, *[Eubacterium]_coprostanoligenes_group*, *Clostridia_UCG-014*, and *Lachnospiraceae* were significantly decreased, and the *Tannerellaceae*, *Bacteroidaceae*, and *Clostridia vadinBB60* groups were significantly increased in the OVA mice (Figure 5K).

### 2.5. Altered Serum Metabolic Profile in OVA Mice

**Figure 6:**
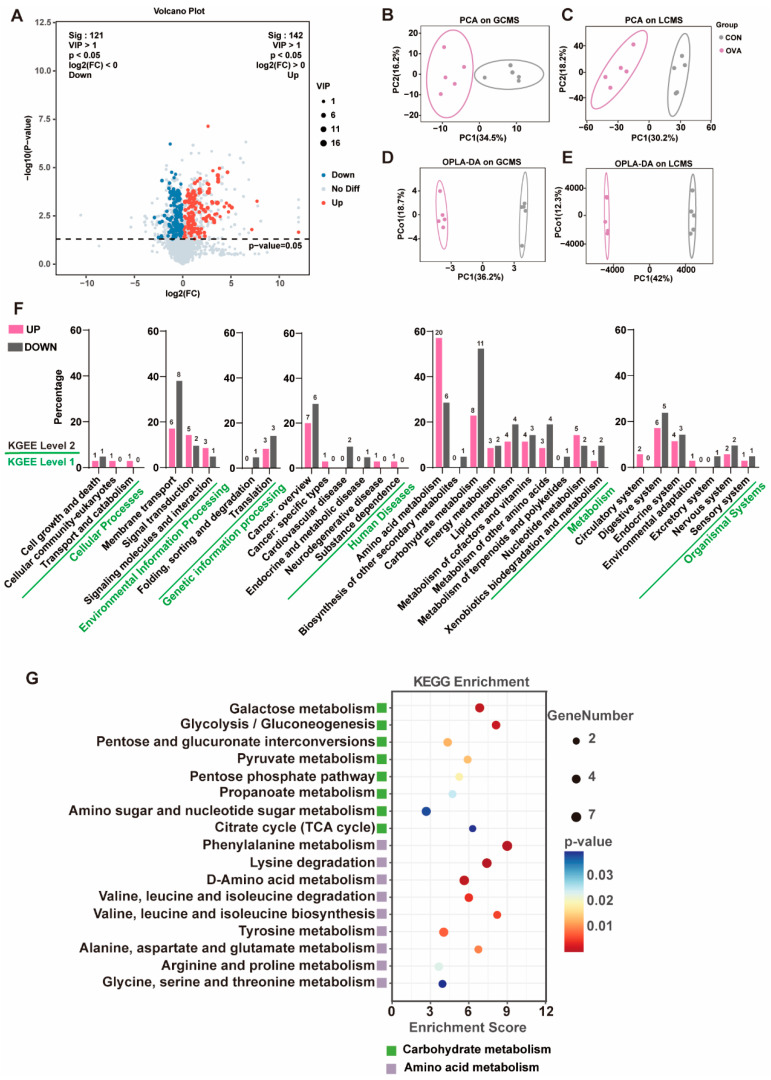
Serum metabolite differences between the OVA group and CON group. (**A**) The Volcano plot of serum metabolites showed 142 significantly upregulated metabolites and 121 significantly downregulated metabolites. (**B**–**E**) Unsupervised PCA presents differences in serum metabolites of 2 groups assessed by (**B**) GCMS and (**C**) LCMS. Supervised PCA analysis (OPLS-DA) also showed differences in serum metabolism groups between the OVA group and CON group assessed by (**D**) GCMS and (**E**) LCMS. (**F**) The involved pathway enriched by significantly differential metabolites in KEGG level 1 and level 2. (**G**) The KEGG enrichment in level 3 includes carbohydrate metabolism and amino acid metabolism.

In global metabolomic profiling, a total of 3442 metabolites were detected, and 263 differentially expressed metabolites with *p* < 0.05 and variable importance of projection values (VIP) > 1 were identified (Figure 6A). PCA exhibited a clear discrepancy in the serum metabolic profiles between the OVA and control mice (Figure 6B,C). Likewise, the Orthogonal Projections to Latent Structures Discriminant Analysis (OPLS-DA) model, which reduces model complexity and enhances interpretability, showed significant clustering between the control and OVA mice (Figure 6D,E), with a good ability to distinguish between the different serum samples (LC-MS/MS, R^2^X = 0.658, R^2^Y = 0.999, Q^2^ = −0.367; GC-MS/MS, R^2^X = 0.634, R^2^Y = 0.998, Q^2^ = −0.298). Based on the KEGG database, a pathway enrichment analysis on the differential metabolites was conducted. The results in Figure 6F show that the differential metabolites involved 31 pathways (level 2), and most of them were concentrated in metabolism (level 1), particularly amino acid and carbohydrate metabolism (level 2). In amino acid and carbohydrate metabolism, compared to the control group, 17 pathways of level 3 were altered in the OVA group (*p* < 0.05) (Figure 6G).

### 2.6. Correlation Between Altered Serum Metabolites and Gut Microbiota

**Figure 7:**
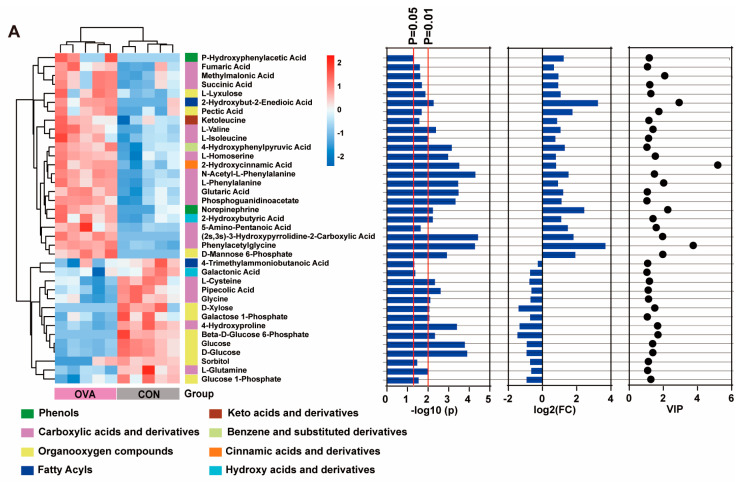

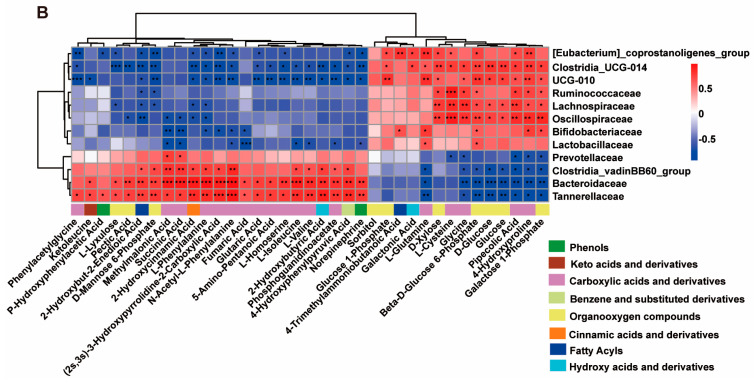
(**A**) Heatmap obtained by Pearson’s hierarchical clustering of metabolites selected from carbohydrate metabolism and amino acid metabolism based on *p* value < 0.05. (**B**) Correlation between different gut microbiota and serum metabolites. * *p* < 0.05, ** *p* < 0.01 and *** *p* < 0.001.

The differential metabolites in amino acid metabolism and carbohydrate metabolism between the two groups were further selected for hierarchical Pearson’s clustering. Consistent with the results of PCA and OPLS-DA, hierarchical Pearson’s clustering analysis also showed significant disparities between the control and OVA mice, and most of the altered metabolites were carboxylic acids and derivatives (Figure 7A). Some of those metabolites, including methylmalonic acids, succinate, phenylalanine, N-acetyl-L-phenylalanine, isoleucine, glutaric acids, valine, phenylacetyl glycine, cysteine, glutamine, and glycine, play important roles in immunity homeostasis and neuronal function. To explore the possible pathway of the reciprocity between gut microbiota and metabolism, Pearson’s correlation between differential metabolites in the serum and differential OTUs at the family level were analyzed (Figure 7B). A highly significant correlation was presented between carboxylic acids and derivatives and the 12 differential OTUs, among which 10 of them are described in Figure 5E and the other 2 are *Prevotellaceae* and *UCG-010* (*p* < 0.05).

## 3. Discussion

Our study first systemically evaluates the neurobehaviors in OVA-sensitized FA mice and explores the potential mechanism in the gut microbiota–brain axis via muti-omics. We observed anxiety, depression, repetitive behavior, and impaired social behavior and attention in food allergy mice, and neuronal damage which might be caused by microglia activation and pro-inflammatory cytokine dysregulation in the PFC of food allergy mice. The disruption in amino acid and carbohydrate metabolism associated with gut microbiota dysbiosis after food allergen exposure could be responsible for the impaired behaviors in food allergy mice.

Previous studies mainly reported the behavioral abnormalities in cow’s milk allergy mice [26,27,28,29]. Although OVA as the major allergen of egg whites has been widely used in a well-established food allergy mouse model [25], the behavioral changes in OVA-induced food allergy mice have been scarcely examined. The current study confirmed that OVA-induced food allergy mice had the same impaired behaviors, like anxiety-, depression- and ASD-like behavior. Consistent with prior studies, in our study, OVA mice also showed a reduced social interaction [27,28,29]. In addition, the social novelty preference was first evaluated via a three-chamber test, with a damaged social interest in FA mice. Likewise, our study first used the Barnes maze to assess cognitive function change in food allergy mice, particularly the long-term retention of spatial memory. In previous studies, food allergy mice showed a declined short-term working memory, which was measured with alternation tasks [26,27]. To our surprise, we did not detect significant differences in our study. Whether our result is due to the limited duration exposure to allergens needs further study to elucidate this. In addition, a decreased attention level was also observed in our FA mice, which might be attributed to the multiple cognitive functions involved in attention, such as memory, discrimination, and orientation. Of note, our research was the first one to report the impaired attention in FA mice. Taken together, behavioral alterations in food allergy mice were extensive, including emotion, sociability, social novelty preference, short-term working memory, and attention. The most model of food allergy is isomorphic murine models (in which the symptoms and treatments are shared); these models as well as the OVA-induced food allergy mice model are also important and convincing in providing insights into the mechanisms, etiology, and potential therapy for food allergies, particularly when working in synergy with human studies [30].

The polarization of microglia and the level of pro-inflammatory cytokines TNF-α, IL-1β, and IL-6 have been widely used to assess neuroinflammation in Alzheimer’s disease, major depression, ASD, and ADHD [31,32,33,34]. As the highest stage of neural integration, the PFC is the cortical region receiving input from the largest number of brain regions and projecting to more brain regions than the other cortical regions, positioning it ideally to coordinate a wide range of neuronal processes [35]. The inflammation and microglial activation in the PFC are also correlated with a variety of behaviors, including anxiety-like [36], depression-like [37], and ASD-like [38] behaviors and attention [39]. Consistent with these prior data [26,40], our study also observed that neuroinflammation consisting of activated microglia and dysregulated pro-inflammatory cytokines occur in the prefrontal cortex of food allergy mice. Dysregulated cytokines not only worsen neuroinflammation by activating microglia directly, but also compromise blood–brain barrier integrity, allowing peripheral immune cells and inflammatory mediators to infiltrate the brain, further amplifying neuroinflammation and neuronal damage [41]. A study demonstrated that only the level of TNF-α was increasing in the cerebral cortex of food allergy mice; the levels of IL-1β and IL-6 were not changed [42]. In the current study, the three cytokine levels, however, were all elevated in the prefrontal cortex. A potential explanation for this discrepancy might be the fact that FA causes region-specific changes in cytokine concentrations [43]. According to previous findings, microglia often damage the neurons by switching their phenotype from a beneficial anti-inflammatory state (M2 phenotype) to a deleterious pro-inflammatory state (M1 phenotype) [44]. In our study, neural damage was also observed in FA mice, which is likely induced by elevated pro-inflammatory cytokines and the polarization of microglia towards the pro-inflammatory phenotype.

In agreement with prior studies, our study also confirms that gut microbiota dysbiosis exists in food allergy mice with abnormal behaviors. At the phylum level, we observed a significant reduction in the Firmicutes/Bacteroidota (F/B) ratios in the OVA group, which was reported in allergic mice [20] and infants [45]. This index also correlated with inflammation and metabolic dysregulation [46,47]. We further analyzed the family level. An increased abundance of *Bacteroidaceae* and *Tannerellaceae* was detected in food allergy mice, which are considered harmful. This elevated abundance of *Bacteroidaceae* is correlated with intestinal ferroptosis and Th2-type systemic inflammation [48]. *Tannerellaceae*, which secretes endotoxins in the gut [49], was positively correlated with pro-inflammatory cytokine TNF-α [50]. In contrast, *Lachnospiraceae*, *Clostridia,* and *Eubacterium_coprostanoligenes*_group, the SCFA-producing bacteria, perform crucial roles in gastrointestinal health, have anti-inflammatory effects, and aid in the development of allergic diseases [51,52,53,54]. The association between *Ruminococcaceae* and indole metabolites is also one of the key factors in maintaining the integrity of the intestinal barrier and regulating the imbalance of Th2/Th1 proportion [55,56,57]. *Bifidobacteriaceae* [58,59] and *Lactobacillaceae* [20], the regulators of amino acid metabolism, effectively alleviated gastrointestinal symptoms by mitigating intestinal epithelial cell damage, reducing allergic inflammation by changing the Th2/Th1 balance toward a dampened Th2 response [60,61,62]. Furthermore, amino acid metabolism, indole metabolism, and SCFA profoundly influence the neurobehaviors and brain function [63,64,65].

Disrupted metabolites and gut microbiota have been reported in individuals with food allergy [14,66] as well as neuropsychiatric disorders [67]. Likewise, our Spearman analysis shows that the serum metabolites were significantly correlated with the balance of the gut microbiota, and a distinctly metabolic signature existed. In our study, the significant alteration of metabolites between the two groups was mainly concentrated in amino acid and carbohydrate metabolism, including carboxylic acids or derivatives involving neuroinflammation or neural damage. Previous investigations mentioned that the accumulation of methylmalonic acids in the body had the potential to induce NH^4^+ surplus and neuron CoQ10 deficiency, which might be the trigger for microglia activation, neuron apoptosis, and neurological dysfunction [68,69]. Increased succinate not only led to increased type 2 innate lymphoid cells and an enhanced type 2 inflammatory response in the small intestine [70], but also induced neuroinflammation and neuronal damage in the PFC [71]. Phenylalanine, N-acetyl-L-phenylalanine, and isoleucine were reported to be associated with Alzheimer’s disease in mouse models, and the peripheral accumulation of these metabolites induced by gut microbiota could stimulate microglia activation, contributing to neuroinflammation and neuropathological changes [72,73]. Other studies showed that the exposure of neurons to glutaric acids and quinolinic acids can create synergistic scenarios, inducing neurotoxic effects on cell viability via the stimulation of oxidative stress [74]. Taken together, most carboxylic acids or derivatives altered in food allergy mice could promote microglia activation and/or neural damage in the end, which contributes to the neurobehavioral abnormalities. Previous metabolomic analysis reported that elevated histidine, valine, phenylacetyl glycine, and tyrosine and decreased cysteine contributed to allergic inflammatory reactions [75,76]. As neurotransmitters, glutamic acids and glycine could drive various behaviors, such as social play behaviors [77], aggressive behaviors [78], exploratory activity, and anxiety- and depression-like behaviors [79,80,81]. All the changes in metabolites mentioned above have been observed in the present study, implying that the regulation of carbohydrate or amino acid metabolic pathways by balancing the gut microbiota could have a positive effect on the overall behavioral phenotype. In addition, intervention with specific taxa or metabolites should be conducted to identify and confirm specific pathways in future.

Several epidemiological studies have reported the association between neurobehavioral changes and allergic conditions. For example, in a nationally representative sample, Xu et al. found a remarkable and positive correlation of food allergy with ASD [12] and ADHD [11]. On the one hand, it is well known that individuals with food allergy exhibited gut dysbiosis, and their anaphylactic symptoms could be improved after microbiota therapy, such as probiotics [14]. On the other hand, emerging studies show the association and causality between gut microbiota and various neurodevelopmental disorders. For example, children with ASD showed a significantly higher abundance of *Bacteroides* and *Clostridium* and a lower percentage of *Bifidobacterium* [82]. Furthermore, fecal microbiota from ASD patients could induce ASD-like behaviors in mice, impair the amino acid metabolism, and alter the expression of ASD-relevant genes in the brain, while the administration of specific microbial-derived amino acid metabolites could improve behaviors in a mouse model [83,84]. Another clinical study by Kang DW et al. found that extended fecal microbiota transplants altered the gut microbiota composition and improved both gastrointestinal and behavioral symptoms in children with ASD and comorbid gut problems [85]. Thus, it is reasonable to hypothesize a common potential microbiota- or metabolite-targeted therapy for both FA-related gastrointestinal symptoms and neurobehavioral abnormalities.

We have to admit that there are some limitations in our study. Firstly, we did not consider the sex difference and some variable control (set up additional groups like a sensitization-only group, challenge-only group, and passive systemic group) when we established the food allergy mice model, which might further influence the findings in behavioral changes or mechanism exploration [86,87]. Secondly, the hippocampus and amygdala are also associated with memory and anxiety, but we only focused on the PFC. Thirdly, we did not perform correlation analysis between the behavioral index and microbiota/metabolites, since the batch of food allergy mice in each research part was different. Lastly, compared to the 16s we employed in this study, metagenome sequencing is a more suitable and state-of-the-art tool to comprehensively investigate the gut microbiota and related functions at species or strain levels. These flaws will be modified in our further study.

In conclusion, wide neurobehavioral alterations were observed in OVA-induced FA mice where neuroinflammation and neuronal damage occurred in the PFC, which is possibly attributed to the dysregulation of carboxylic acids and derivatives resulting from gut microbiota dysbiosis after allergen exposure. Although we have found that the gut microbiota–brain axis played a vital role in the pathogenesis of neurobehavioral abnormalities in OVA-induced food allergy mice, the specific mechanism has not been explored. In fact, a variety of potential mechanisms between the gut microbiota–brain axis and diseases have been reported, but the causality is still unclear. Additionally, gut microbiota may be a key contributor to the prevention, development, treatment, and management of many diseases, including food allergy and neurodevelopment disorders, and enormous challenges remain in the successful translation to the bench side since existing animal models are limited in the replication of human disease. Therefore, the role of microbiota in diseases, like food allergy and neurodevelopment, needs continuous attention as well as more preclinical or clinical studies to verify this.

## 4. Materials and Methods

### 4.1. Animals

Three- to four-week-old C57BL/6J male mice were purchased from Shanghai Jihui Laboratory Animal Technology Co., Ltd. (Shanghai, China) (certificate number: SCXK 2022-0009). Mice were randomly divided into OVA and control groups and housed under a 12 h light/12 h dark cycle in a specific-pathogen-free environment, fed by OVA-free food and water ad libitum.

### 4.2. Experimental Protocol

The experimental protocol is displayed in Figure 1. Briefly, OVA mice were sensitized with 100 μg of ovalbumin (OVA grade V, Sigma-Aldrich, Burlington, MA, USA, A5505) and 2 mg of aluminum adjuvant (Sigma-Aldrich, Burlington, MA, USA, 239186) via intraperitoneal (i.p.) injection on days 0, 7, and 14, and then the mice were challenged every other day with 60 mg of OVA by intragastric gavage (i.g.) from day 21 to day 39. Control mice were only sham sensitized with 2 mg of aluminum adjuvant by i.p. injection and challenged with PBS. The body weights were recorded throughout the experiment, and physical symptoms were observed and scored according to the rules listed in Table S1 within 60 min after the challenges. Rectal temperature was also obtained after the challenge.

### 4.3. Behavioral Experiments

Behavioral tests including the open-field test (OFT), elevated plus maze (EPM), forced swimming test (FST), self-grooming, marble burying test (MBT), three-chamber sociability test (TST), non-selective, non-sustained visual attention test (NNAT), and Barnes maze test were conducted during the light phase of the illumination cycle. On the test day, mice were transported to the testing room and left in their home cages for at least 1 h before the tests. EthoVision XT 15 software (Noldus Information Technology Inc. Wageningen, The Netherlands) was applied to analyze every behavioral index.

#### 4.3.1. OFT

An open field with a dimension of 35 × 35 cm surrounded by 50 cm high walls was used. Mice were individually placed in the same area of the enclosure outside of the central zone and allowed to freely explore for 10 min. The total time spent in the central zone, the average speed, and the distance traveled were recorded to objectively assess anxiety-like behavior as well as overall activity levels.

#### 4.3.2. EPM

The apparatus consists of two open arms (30 cm × 6 cm × 0 cm) and two perpendicular closed arms (30 cm × 6 cm × 15 cm) extending from a central platform. The entire maze was elevated approximately 80 cm from the floor. Mice were placed in the central zone and allowed to explore the maze freely for 5 min. The number of entries into each arm and the total time spent in each arm were used to evaluate anxiety level. Reductions in the percentage of open arm exploration (time in the open arm divided by the total time in the maze) were interpreted as increased anxiety.

#### 4.3.3. FST

Mice were placed into a 4 L cylindrical water bath filled with 3 L of water at 24–26 °C under bright light conditions. The test lasted for 6 min and the time spent immobile was measured. Immobility was defined as no movement at all or only minor movements necessary to keep the nose above the water. Higher degrees of depression are indicated with more time spent immobile.

#### 4.3.4. Self-Grooming

Mice were habituated to an empty cage for 10 min and then timed for an additional 20 min for spontaneous self-grooming behavior. Grooming was defined as the time spent licking paws, washing the nose and face, or scratching fur with any foot. Grooming time was recorded with a stopwatch by two examiners who were blind to the treatment conditions. The increased time spent grooming was interpreted as a high level of repetitive behaviors [88].

#### 4.3.5. MBT

Mice were habituated for 10 min in a clean Plexiglas cage filled with a 5 cm thick layer of sawdust. Following habituation, 20 glass marbles were gently laid out in five rows of 4 marbles placed equidistantly apart. Mice were then allowed to explore for 10 min. Only marbles covered by 75% or more bedding were counted as buried, and the number of marbles buried was recorded [89].

#### 4.3.6. TST

The apparatus was a yellow plexiglass box (60 cm length, 40 cm width) divided into three chambers (20 × 40 cm) by transparent plexiglass walls. A mouse subject was first habituated to the full empty arena for 10 min and then entered 2 phases: sociability (stranger 1 vs. empty) and social novelty (stranger 1 vs. stranger 2). In Session 1 (sociability), a novel same-sex mouse was placed in one of the small wire cages (Stranger 1), while the other remained empty (Empty). The experimental mouse was allowed to freely explore the three chambers again for 10 min. In Session 2 (social novelty), Empty was replaced with Stranger 2, and the test mouse was allowed another 10 min of free exploration in this session. The sides where Empty, Stranger 1, and Stranger 2 were placed were randomly assigned. The time spent exploring each cage was calculated to represent social behaviors.

#### 4.3.7. NNAT

Modified NNAT was used to test the interest in novel object exploration and assess the attention levels in mice [90,91]. Four objects were fixed onto the corners of the bottom of the square box. They were all about 1.5 cm in diameter, of the same color (golden) and texture, but different in shape. After the 10 min familiarization session, the contact time between the mice and the “new object” was recorded for 10 min in the test session. The attention level is the percentage of time that they contact with “new objects” divided by the total contact time between mice and all four objects.

#### 4.3.8. Barnes Maze Test

The Barnes maze is used to evaluate long-term spatial memory in mice [92]. The behavioral test consists of five days of training and one day of testing. The Barnes circular maze is a white platform with a diameter of 122 cm and a height of 70 cm. A total of 20 holes are evenly placed at the edge of the disk, of which only 1 hole is equipped with an escape box. A loud buzzer sound of 80 dB was provided as stimuli, forcing the mice to explore and hide in the box. Additionally, visual cues were stuck on the walls of the laboratory room. The mice were trained twice a day during the training period. If the mice did not enter the escape box within 300 s, they were guided into it and maintained there for another 30 s. The tests were conducted after the training phase and each mouse was active for 300 s. The time when mice entered the escape box and spent time in the target quadrant was counted.

### 4.4. Enzyme-Linked Immunosorbent Assay (ELISA)

Two hours after the allergen challenge, mice were anesthetized with an i.p. injection of pentobarbital and an inhalation of isoflurane, and then they were sacrificed. For serum preparation, blood was collected in a 1.5 mL centrifuge tube and kept at room temperature for 2 h before centrifuging at 3500× *g* for 10 min at 4 °C. After sacrifice, the dissected prefrontal cortex was lysed using a motorized tissue cutter in lysis buffer (Absin, Shanghai, China, abs9225) and freshly added protease inhibitor cocktail (Thermo Fisher, Waltham, MA, USA, 1861281). The supernatants were collected after centrifuging at 12,000× *g* for 20 min at 4 °C, and their protein concentrations were measured using a BCA protein assay kit (Beyotime, Shanghai, China, P0010). The levels of OVA-IgE (Chondrex, Woodinville, WA, USA, 3010), OVA-IgG1 (Chondrex, Woodinville, WA, USA, 3013), and histamine (Bertin, Paris, France, A05890) in serum were detected using ELISA kits according to the manufacturer’s instructions. The cytokine levels in serum and the brain were measured by Luminex^®^ Discovery Assay multiplex kits (R & D Systems, Wiesbaden, Germany, LXSAMSM).

### 4.5. Immunofluorescence Staining and Histopathological Assessment

Mice for immunofluorescence were subjected to whole-body perfusion via a right-heart catheter with PBS and then with 4% paraformaldehyde. The whole brains were dissected out from the cranial cavity and coronal slices were prepared by a Leica CM1950 cryostat microtome (Leica, Wetzlar, Germany). Prefrontal cortex slices were selected according to a brain atlas and incubated in PBS containing 10% normal goat serum (Absin, Shanghai, China, abs933) and 0.1% Triton X-100 (Sigma-Aldrich, Burlington, MA, USA, T8787) at room temperature for 2 h. They were then treated with primary antibodies Iba-1 (1:500 dilution; Wako, Japan, 019-19741) overnight at 4 °C in PBS and washed with PBS. The signals were visualized using the Alexa Fluor 594-conjugated secondary antibodies (1:500; Thermo Fisher, Waltham, MA, USA, A150080). To evaluate neuronal and intestinal damage, hematoxylin and eosin (HE) (Solarbio Science and Technology, Beijing, China) and Nissl staining (Solarbio Science and Technology, Beijing, China) were carried out according to the manufacturer’s instructions. The images of the immunofluorescence were captured with a Leica confocal microscope.

### 4.6. Fecal Microbiome Analysis

DNA was purified from fecal specimens using a ZymoBIOMICS DNA kit (Zymo Research, Irvine, CA, USA). The 16S ribosomal DNA (V3-V4 region) of the microbial DNA extracted from the stool sample was amplified with primers (343F 5′-TACGGRAGGCAGCAG515-3′; 798R 5′AGGGTATCTAATCCT806-3′). After PCR, the agarose gel containing target DNA fragments was cut for purification using the DNA Gel/PCR Purification Miniprep Kit (BW-DC3511, Beiwo Meditech Co., Ltd., Hangzhou, China) according to the manufacturer’s instructions. Taxonomic classification was performed using the plugin q2-feature-classifier64, a taxonomic classifier plugin for the QIIME 2 microbiome analysis platform (https://qiime2.org/), which makes similar calculations using a scikit-learn naive Bayes classifier. Finally, taxonomy was assigned to filtered amplicon sequence variants (ASVs) using a pretrained QIIME2-compatible SILVA version 132 database, with 99% identity for the bacteria and representative sequences.

### 4.7. Metabolome Analysis

The serum metabolites were analyzed using LC-MS/MS&GC-MS/MS by Shanghai Luming Biological Technology Co., Ltd. (Shanghai, China). UPLC-Q-TOF/MS (ACQUITY UPLC I-Class, Waters, MA, USA) and ESI-QTOF/MS (QE plus, Thermo Fisher Scientific, Waltham, MA, USA) were used. The LC-MS/MS analysis was performed by an ACQUITY UPLC I-Class plus (Waters Corporation, Milford, CT, USA) fitted with a Q-Exactive plus mass spectrometer (Thermo Fisher Scientific, Waltham, MA, USA), and GC-MS/MS was performed by an Agilent 7890B gas chromatography system coupled to an Agilent 5977B MSD system (Agilent Technologies Inc., Santa Clara, CA, USA). A two-tailed Student’s *t*-test was further used to verify whether the metabolites of difference between groups were significant. Differential metabolites were selected with VIP values greater than 1.0 and *p*-values < 0.05. The enriched pathway analysis of changed metabolites was performed using the KEGG database (https://www.kegg.jp/kegg/pathway.html, accessed on 30 June 2024).

### 4.8. Statistical Analysis

All data are presented as the mean ± SEM. The statistical difference in mean values between two groups was determined by the *t* test. A *p* value < 0.05 was considered statistically significant. Graphs and statistical analyses were all made using Graph Pad Prism 7.0 software (Graph Pad, La Jolla, CA, USA).

## Data Availability

The datasets from the current study are available from the corresponding author upon request.

## Supplementary Materials

The following supporting information can be downloaded at https://www.mdpi.com/article/10.3390/ijms26104760/s1.

## Abbreviations

The following abbreviations are used in this manuscript:

FA	Food allergy
OVA	Ovalbumin
PFC	Prefrontal Cortex
OFT	Open-field test
EPM	Elevated plus test
FST	Forced swimming test
MBT	Marbles buried test
TST	Three-chamber social test
NNAT	Non-selective, non-sustained visual attention test
