# Supplementary material for: OVA-Induced Food Allergy Leads to Neurobehavioral Changes in Mice and the Potential Role of Gut Microbiota and Metabolites Dysbiosis
Source: Int J Mol Sci. 2025 May 16;26(10):4760. doi: 10.3390/ijms26104760 (PMC12111871; doi:10.3390/ijms26104760)
Supplement: Supplementary file 1 [file ijms-26-04760-s001.zip › ijms-3595669-supplementary.pdf]

Supplementary Table S1. Anaphylaxis scoring scales.

0	No reaction/clinical symptoms
1	Scratching and rubbing around the nose and head
2	Puffiness around the eyes and mouth, pilar erecti, reduced activity
3	Wheezing, labored respiration and cyanosis around the mouth and tail
4	No activity after prodding or tremor and convulsion
5	Death

Supplementary Figure S1

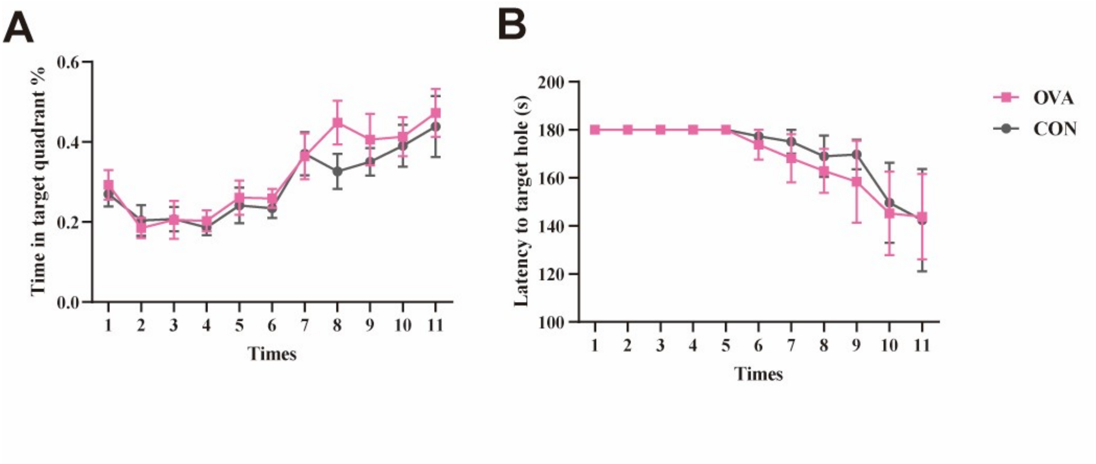

Supplementary Figure S1. Barnes maze test. Control group and OVA group mice spent similar time in (A) target quadrant and (B) latency to target hole.
